# A new species of *Gracixalus* (Anura, Rhacophoridae) from Yunnan, China

**DOI:** 10.3897/zookeys.851.32157

**Published:** 2019-06-03

**Authors:** Guohua Yu, Hong Hui, Jian Wang, Dingqi Rao, Zhengjun Wu, Junxing Yang

**Affiliations:** 1 Key Laboratory of Ecology of Rare and Endangered Species and Environmental Protection (Guangxi Normal University), Ministry of Education, Guilin 541004, China; 2 Guangxi Key Laboratory of Rare and Endangered Animal Ecology,; 3 College of Life Science, Guangxi Normal University, Guilin 541004, China; 4 State Key Laboratory of Genetic Resources and Evolution, Kunming Institute of Zoology, Chinese Academy of Sciences, Kunming, Yunnan 650223, China; 5 College of Life Science and technology, Honghe University, Mengzi, Yunnan 661199, China

**Keywords:** *Gracixalusyunnanensis* sp. n., taxonomy, Rhacophoridae, southwestern China

## Abstract

A new species of the genus *Gracixalus*, *Gracixalusyunnanensis***sp. n.**, is described based on a series of specimens collected from southwestern and southern Yunnan, China. This species is distinguished from all other known congeners by a combination of the following characters: relatively small body size in adult males (SVL 26.0–34.2 mm); dorsal surface yellow brown or red brown; distinctive conical asperities on dorsum; males with an external subgular vocal sac and linea masculina; throat, chest, and belly nearly immaculate; venter surface orangish with yellow spots, semi-transparent; snout rounded; supratympanic fold distinct; iris bronze; lack of white patch on temporal region; tibiotarsal projection absent; sides of body nearly smooth with no black blotch; finger webbing rudimentary; and toe webbing formula I1.5–2II1.5–2.7III.5–3IV2.5–1.5V. Genetically, the new species diverges from its congeners by 2.2%–14.1% (uncorrected p-distance) and is closest to *G.guangdongensis*. However, the new species can morphologically be separated from *G.guangdongensis* by distinctive conical tubercles on dorsum (versus absent), lateral surface nearly smooth with no black blotches on ventrolateral region (versus lateral surface rough, scattered with tubercles and black blotches on ventrolateral region), snout rounded (versus triangularly pointed), iris bronze (versus iris brown), and ventral surface orangish (versus throat and chest creamy white and belly light brown).

## Introduction

The genus *Gracixalus* Delorme, Dubois, Grosjean & Ohler, 2005 is known from southern and southwestern China, Vietnam, Laos, Thailand, and Myanmar and contains 16 species including *G.ananjevae* (Matsui & Orlov, 2004), *G.carinensis* (Boulenger, 1893), *G.gracilipes* (Bourret, 1937), *G.guangdongensis* Wang, Zeng, Liu & Wang, 2018, *G.jinggangensis* Zeng, Zhao, Chen, Chen, Zhang & Wang, 2017, *G.jinxiuensis* (Hu, in Hu et al. 1978), *G.lumarius* Rowley, Le, Dau, Hoang & Cao, 2014, *G.medogensis* (Ye & Hu, 1984), *G.nonggangensis* Mo, Zhang, Luo, Zhou & Chen, 2013, *G.quangi*, Rowley, Dau, Nguyen, Cao & Nguyen, 2011, *G.quyeti* (Nguyen, Hendrix, Bohme, Vu & Ziegler, 2008), *G.sapaensis* Matsui, Ohler, Eto & Nguyen, 2017, *G.seesom* Matsui, Khonsue, Panha & Eto, 2015, *G.supercornutus* (Orlov, Ho & Nguyen, 2004), *G.tianlinensis* Chen, Bei, Liao, Zhou & Mo, 2018, and *G.waza* Nguyen, Le, Pham, Nguyen, Bonkowski & Ziegler, 2013 (Frost 2018). Of the 16 members of *Gracixalus*, ten were discovered in last decade (Nguyen et al. 2008, Rowley et al. 2011, Mo et al. 2013, Nguyen et al. 2013, Rowley et al. 2014, Matsui et al. 2015, Matsui et al. 2017, Zeng et al. 2017, Chen et al. 2018, Wang et al. 2018), indicating that species diversity of *Gracixalus* was very poorly understood in the past. Moreover, recent phylogenetic analyses (Matsui et al. 2017, Chen et al. 2018) showed that there are still several unnamed distinct lineages in the group of *G.jinxiuensis*, indicating that species richness of *Gracixalus* remains underestimated.

During recent fieldworks in Yunnan, China, we collected some specimens of a small-sized tree frog, which morphologically can be assigned to the genus *Gracixalus* by the presence of intercalary cartilage between terminal and penultimate phalanges of digits, tips of digits enlarged to discs bearing circummarginal grooves, vomerine teeth absent, inner (first and second) and outer (third and fourth) fingers non-opposable, and an inversed Y-shaped dark brown marking on dorsum (Fei 1999, Rowley et al. 2011, Chen et al. 2018), but morphologically and genetically can be distinguished from all recognized species of genus *Gracixalus*. Thus, we describe these specimens as a new species of *Gracixalus*.

## Materials and methods

### Sampling

**Figure 1.**
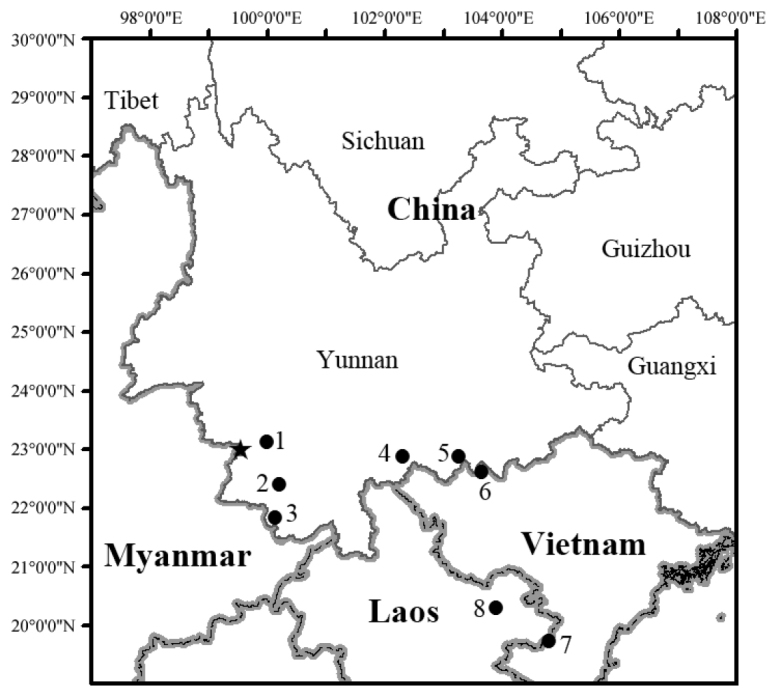
Map showing collection sites of *Gracixalusyunnanensis* sp. n. Star indicates the type locality (Xuelin) and circles indicate Fudong Township (**1**), Fazhanhe Township (**2**), Bada Township (**3**), Mt. Huanglian (**4**), Jinping (**5**), Lao Cai (**6**), Nghe An (**7**), and Houapan (**8**), respectively. Sequences of samples from sites 5–8 came from previous studies.

Specimens were collected during fieldworks in Menghai County, Lancang County, and Lvchun County of Yunnan, China in 2014 to 2018 (Fig. 1). They were fixed and then stored in 80% ethanol after taking photos. Liver tissues were preserved in 99% ethanol. Specimens were deposited at the Kunming Institute of Zoology (KIZ), Chinese Academy of Sciences and Guangxi Normal University (GXNU).

### Morphology

Morphometric data were taken using digital calipers to the nearest 0.1 mm. Morphological terminologies follow Matsui et al. (2017) and Wang et al. (2018). Measurements include:

**SVL** snout-vent length (from tip of snout to vent);

**HL** head length (from tip of snout to rear of jaws);

**HW** head width (width of head at its widest point);

**SL** snout length (from tip of snout to anterior border of eye);

**IND** internarial distance (distance between nares);

**IOD** interorbital distance (minimum distance between upper eyelids);

**UEW** upper eyelid width (maximum width of upper eyelid);

**ED** eye diameter (diameter of exposed portion of eyeball);

**TD** tympanum diameter;

**FHL** forearm and hand length (from elbow to tip of third finger);

**THL** thigh length (from vent to knee);

**TL** tibia length (distance from knee to heel);

**FL** foot length (from proximal end of inner metatarsal tubercle to tip of fourth toe);

**TFL** length of foot and tarsus (from tibiotarsal joint to tip of fourth toe).

Comparative morphological data of other *Gracixalus* species were taken from their original descriptions or re-descriptions (Boulenger 1893, Hu et al. 1981, Ye and Hu 1984, Matsui and Orlov 2004, Orlov et al. 2004, Nguyen et al. 2008, Rowley et al. 2011, Mo et al. 2013, Nguyen et al. 2013, Rowley et al. 2014, Matsui et al. 2015, Matsui et al. 2017, Zeng et al. 2017, Chen et al. 2018, Wang et al. 2018).

### Molecular analyses

Total genomic DNA was extracted from liver tissues. Tissue samples were digested using proteinase K, and subsequently purified following a standard phenol/chloroform isolation and ethanol precipitation. A fragment encoding partial 16S rRNA gene was amplified and sequenced following Yu et al. (2010). All new sequences have been deposited in GenBank under Accession Nos. MK234876–MK234883 (Table 1). Available homologous sequences of *Gracixalus* were obtained from GenBank. *Rhacophorusborneensis* Matsui, Shimada & Sudin, 2013 and *Kurixalusidiootocus* (Kuramoto & Wang, 1987) were selected as outgroups according to Matsui et al. (2017) and sequences of them were also downloaded from GenBank.

**Table 1.** Species used in phylogenetic analysis of this study.

Species	Locality	Voucher no.	GenBank no.
* Rhacophorus borneensis *	Sabah, Malaysia	BORN 22410	AB781693
* Kurixalus idiootocus *	Taiwan, China	KUHE 12979	AB933306
* Gracixalus ananjevae *	Nghe An, Vietnam	VNMN 03012	JN862546
* Gracixalus gracilipes *	Ha Giang, Vietnam	AMNH A163897	DQ283051
	Pingbian, Yunnan, China	060821196Rao	GQ285668
	Lao Cai, Vietnam	AMS R 177672	KT374014
* Gracixalus guangdongensis *	Hunan, China	CIB HN201108200	LC011936
	Guangdong, China	SYS a004902	MG520193
	Guangdong, China	SYS a005750	MG520197
* Gracixalus jinggangensis *	Mt. Jinggang, Jiangxi	SYS a003186	KY624587
* Gracixalus jinxiuensis *	Jinxiu, Guangxi, China	SYS a002182	KY624584
	Jinxiu, Guangxi, China	SYS a002183	KY624585
	Jinxiu, Guangxi, China	KIZ 060821013	EF564524
	Jinxiu, Guangxi, China	KIZ 061210YP	EU215525
* Gracixalus lumarius *	Kon Tum, Vietnam	AMS R 176202	KF918412
* Gracixalus nonggangensis *	Guangxi, China	NHMG 200910010	JX841318
* Gracixalus quyeti *	Cha Noi, Vietnam	VNUH 160706	EU871428
* Gracixalus quangi *	Nghe An, Vietnam	AMS R173417	JN862539
* Gracixalus sapaensis *	Lao Cai, Vietnam	MNHN 1999.5961	AY880503
	Lai Chau, Vietnam	IEBR 2351	EU871425
	Lao Cai, Vietnam	CIB XM-439	GQ285670
	Lao Cai, Vietnam	KUHE 46401	LC011938
	Lao Cai, Vietnam	KUHE 46402	LC011939
	Lao Cai, Vietnam	MNHN 1999.5966	LC140970
	Lao Cai, Vietnam	VNMN 4211	LC140971
	Lao Cai, Vietnam	VNMN 4212	LC140972
	Lao Cai, Vietnam	VNMN 4358	LC140973
* Gracixalus seesom *	Kanchanaburi, Thailand	KUHE 35084	LC011932
* Gracixalus supercornutus *	Kon Tum, Vietnam	AMS R173887	JN862545
	Gia Lai, Vietnam	AMS R176287	KT374016
* Gracixalus tianlinensis *	Guangxi, China	NHMG 1705015	MH117960
	Guangxi, China	NHMG 1705016	MH117961
* Gracixalus waza *	Cao Bang, Vietnam	IEBR A.2012.2	JX896681
	Cao Bang, Vietnam	VNMN A.2012.2	JX896684
*Gracixalus* sp.	Wenshan, Yunnan, China	03320Rao	GQ285669
*Gracixalusyunnanensis* sp. n.	Houapan, Laos	KUHE 32453	LC011937
	Lao Cai, Vietnam	VNMN 4355	LC140985
	Lao Cai, Vietnam	VNMN 4357	LC140986
	Lao Cai, Vietnam	VNMN 4371	LC140987
	Nghe An, Vietnam	AMS R173454	JN862547
	Jinping, Yunnan, China	KIZ 060821126	EF564525
	Lvchuan, Yunnan, China	GXNU YU000060	MK234876
	Bada, Menghai, Yunnan, China	KIZ 20160216	MK234877
	Xuelin, Lancang, Yunnan, China	KIZ 20160222	MK234878
	Xuelin, Lancang, Yunnan, China	KIZ 20160223	MK234879
	Fudong, Lancang, Yunnan, China	KIZ 20160226	MK234880
	Fazhanhe, Lancang, Yunnan, China	KIZ 20160228	MK234881
	Fazhanhe, Lancang, Yunnan, China	KIZ 20160229	MK234882
	Fazhanhe, Lancang, Yunnan, China	KIZ 20160230	MK234883

Sequences were aligned using MUSCLE with the default parameters in MEGA version 7 (Kumar et al. 2016). Uncorrected pairwise distances between species were calculated in MEGA version 7. The best substitution model was selected using the corrected Akaike Information Criterion (AICc) in jMODELTEST version 2.1.10 (Darriba et al. 2012). Three methods were used to construct phylogeny of the genus *Gracixalus*. Firstly, Bayesian inference (BI) was performed in MRBAYES version 3.2.6 (Ronquist et al. 2012) based on the selected substitution model (TIM2 + I + G). Two runs were performed simultaneously with four Markov chains starting from random tree. The chains were run for 5,000,000 generations and sampled every 100 generations. Convergence and burn-in were checked using the program Tracer version 1.6. (Rambaut et al. 2014) and plot of the generation versus the log likelihood values. The first 25% of the sampled trees were discarded as burn-in and the remaining trees were used to create a consensus tree and to estimate Bayesian posterior probabilities (BPPs). Secondly, maximum likelihood (ML) analysis was conducted in RAXML-HPC version 8.2.10 (Stamatakis 2014) with 1000 rapid bootstrap replicates. Finally, a neighbor-joining (NJ) tree was constructed using PAUP^*^ version 4.0b10 (Swofford 2002) and nodal supports were assessed by 1000 bootstrap replicates.

## Results

**Figure 2.**
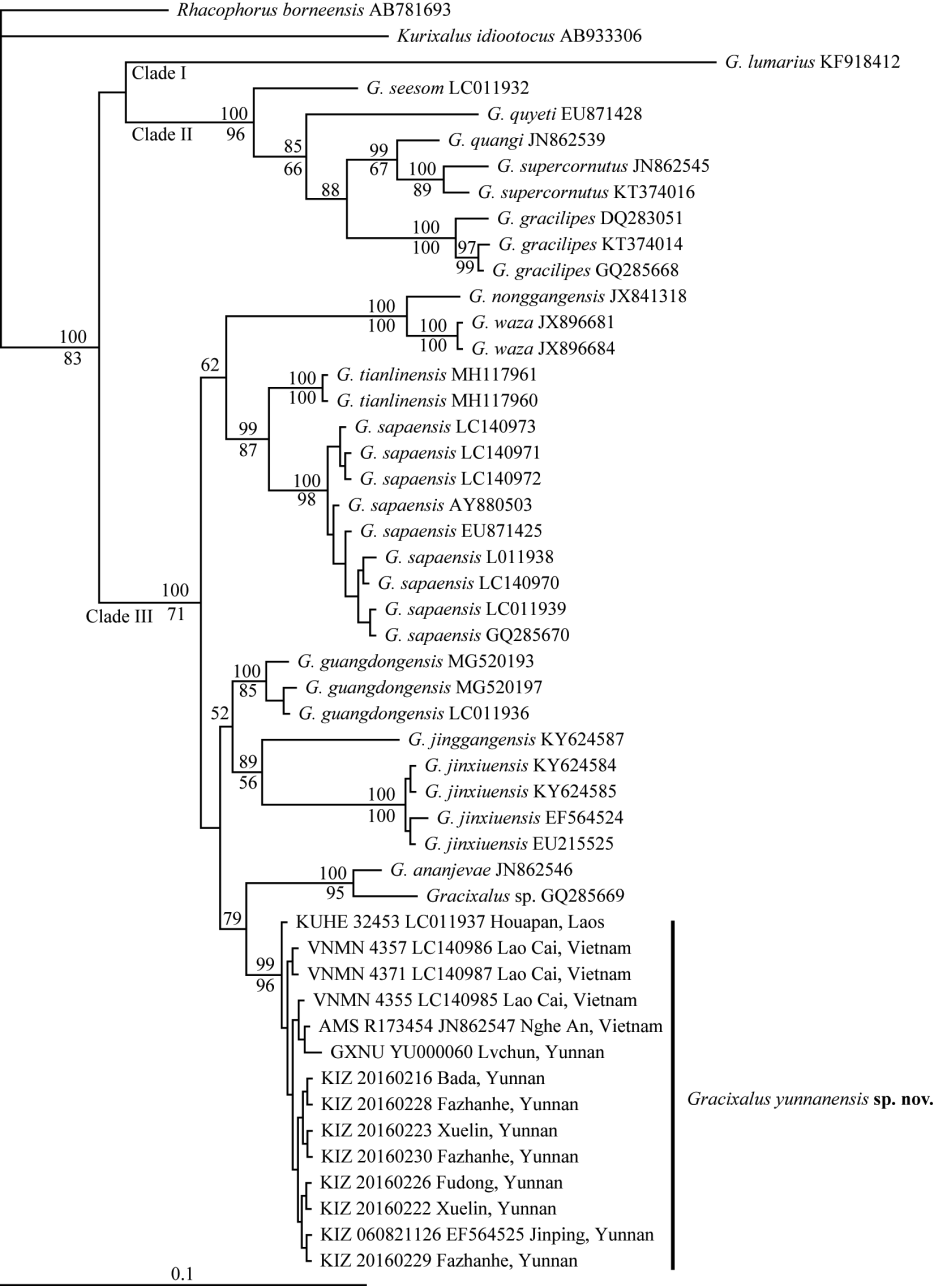
Bayesian phylogram of *Gracixalus* inferred from 543 bp of 16S rRNA gene. Numbers above and below branches are Bayesian posterior probabilities and ML bootstrap values (only values above 50% are shown), respectively.

**Figure 3.**
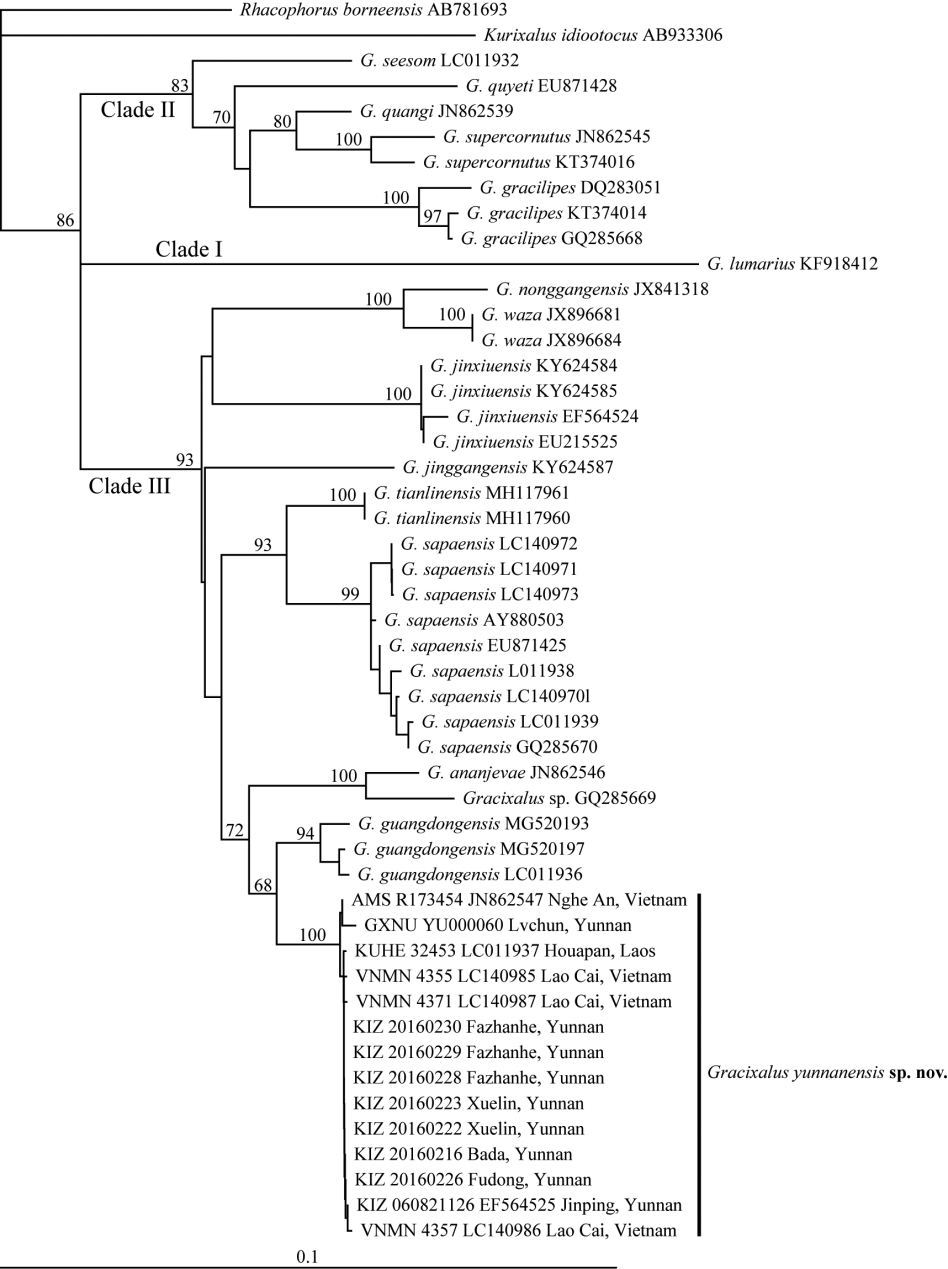
Neighbor-joining (NJ) tree of *Gracixalus* inferred from 543 bp of 16S rRNA gene. Numbers above branches are bootstrap values (only values above 50% are shown).

The obtained alignment of 16S rRNA sequences is 543 bp in length after cutting off both ragged sides. The newly collected samples from Bada, Xuelin, Fudong, Fazhanhe, and Lvchun of Yunnan, China form a distinct lineage together with samples from Houapan of Laos (KUHE 32453), Nghe An (AMS R173454) and Lao Cai (VNMN 4355, 4357, 4371) of Vietnam, and Jinping of Yunnan (KIZ 060821126) that were sequenced by previous studies (Yu et al. 2008, Rowley et al. 2011, Matsui et al 2015, Matsui et al. 2017) (Figs 2, 3). Both Bayesian inference and Maximum likelihood analyses recovered this lineage as the sister to the clade consisting of *G.ananjevae* and *Gracixalus* sp. (GQ285669) with weak support (Fig. 2), whereas the NJ analysis revealed that it is closest to *G.guangdongensis* with weak support (Fig. 3). Average uncorrected pairwise distances (p-distance) between the new species and other species ranged from 2.2% (*G.guangdongensis*) to 14.1% (*G.lumarius*) (Table 2).

Morphologically, these newly collected specimens can be distinguished from *G.guangdongensis* by a series of characters, including distinctive conical tubercles on dorsum (versus absent), lateral surfaces nearly smooth with no black blotches on ventrolateral region (versus lateral surfaces rough, scattered with tubercles and black blotches on ventrolateral region), snout rounded (versus triangularly pointed), iris bronze (versus iris brown), and ventral surface orangish (versus throat and chest creamy white and belly light brown). These specimens also differ from other members of *Gracixalus* in a series of characters. Herein we describe these specimens as a new species.

**Table 2.** Uncorrected p-distances (%) between *Gracixalus* species estimated from 16S rRNA sequences.

	Species	1	2	3	4	5	6	7	8	9	10	11	12	13	14	15	16
1	*Gracixalusyunnanensis* sp. n.	–															
2	* G. ananjevae *	3.9	–														
3	*Gracixalus* sp. (GQ285669)	5.1	2.3	–													
4	* G. sapaensis *	4.7	6.0	6.6	–												
5	* G. quangi *	8.0	9.6	9.5	9.1	–											
6	* G. supercornutus *	9.2	10.9	10.7	10.5	3.0	–										
7	* G. nonggangensis *	7.1	8.8	8.9	7.9	10.5	11.5	–									
8	* G. waza *	6.3	8.7	9.3	7.3	11.6	12.8	2.5	–								
9	* G. lumarius *	14.1	14.5	14.6	15.9	14.3	15.3	16.0	16.0	–							
10	* G. gracilipes *	10.3	11.0	11.3	10.4	5.2	6.2	12.4	13.3	15.0	–						
11	* G. jinxiuensis *	6.0	7.3	7.5	7.2	9.6	10.9	7.7	7.9	16.1	11.4	–					
12	* G. jinggangensis *	5.0	7.3	7.7	6.3	8.0	9.5	7.5	7.5	14.5	11.3	7.0	–				
13	* G. seesom *	8.6	10.4	9.7	8.8	6.0	6.7	10.2	10.1	16.0	6.1	9.6	9.9	–			
14	* G. quyeti *	10.5	11.4	11.0	10.9	6.5	6.1	11.2	12.2	14.0	7.3	10.2	11.1	8.1	–		
15	* G. tianlinensis *	4.3	6.3	6.5	3.0	9.4	10.5	7.2	6.4	14.8	10.3	5.9	6.5	7.8	10.0	–	
16	* G. guangdongensis *	2.2	4.6	5.4	5.0	7.8	9.5	7.2	6.7	14.2	10.1	5.6	5.3	8.3	10.6	4.4	–

### Gracixalus yunnanensis sp. n.

Taxon classificationAnimaliaAnuraRhacophoridae

http://zoobank.org/1D19A62E-B4B2-4EDA-975D-4DCFD58DEDAD

Figs 4 , 5 , 6

#### Type material.

***Holotype.***KIZ 20160222, an adult male, collected at 21:05 on 1 June 2017 by Hong Hui from Xuelin Township, Lancang County, Yunnan Province, China (23°0'39.4"N, 99°31'54"E, 1864 m elevation).

***Paratypes.*** Seven adult males: KIZ 20160223 collected at 21:05 on 1 June 2017 by Hong Hui from the type locality; KIZ 20160216 collected at 21:00 on 7 June 2014 by Hong Hui from Bada Township, Menghai County, Yunnan Province, China (21°50'8.9"N, 100°6'57.8"E, 1870 m elevation); KIZ 20160226 collected at 21:50 on 27 May 2017 by Hong Hui from Fudong Township, Lancang County, Yunnan Province, China (23°7'13.6"N, 99°58'33.9"E, 2166 m elevation); KIZ 20160228–20160230 collected at 21:40–22:15 on 10 June 2017 by Hong Hui from Fazhanhe Township, Lancang County, Yunnan Province, China (22°24'3.4"N, 100°12'4.2"E, 1822 m elevation); and GXNU YU000060 collected at 21:00 on 7 June 2018 by Jian Wang from Mt. Huanglian, Lvchun County, Yunnan Province, China (22°53'N, 102°18'E, 1918 m elevation).

#### Etymology.

The specific epithet *yunnanensis* refers to the distribution of this species in China, Yunnan Province.

#### Diagnosis.

The new species is assigned to genus *Gracixalus* based upon molecular data and the following morphological characters: the presence of intercalary cartilage between terminal and penultimate phalanges of digits, tips of digits enlarged to discs bearing circummarginal grooves, vomerine teeth absent, inner (first and second) and outer (third and fourth) fingers non-opposable, and an inversed Y-shaped dark brown marking on dorsum (Fei 1999, Rowley et al. 2011, Chen et al. 2018). The new species is distinguished from its congeners by a combination of 1) SVL 26.0–34.2 mm in males; 2) dorsal surface yellow brown or red brown; 3) distinctive conical tubercles on dorsum; 4) males with an external subgular vocal sac; 5) throat granular; 6) finger webbing rudimentary; 7) linea masculina, a band of connective tissue between the rectus abdominus muscle and oblique abdominus muscle, present in males; 8) tibiotarsal articulation reaching eye; 9) snout rounded; 10) white patch absent on temporal region; 11) tibiotarsal projection absent; 12) supratympanic fold distinct; 13) ventral surface orangish, nearly immaculate, and semi-transparent; 14) nuptial pads present on finger I; 15) heels overlapping when legs at right angle to body; 16) iris bronze; and 17) body sides nearly smooth with no black blotch.

#### Description of holotype.

Adult male (SVL 29.7 mm); head wider (HW 10.9 mm) than long (HL 9.5 mm); snout rounded, slightly projecting beyond margin of lower jaw in ventral view, rounded in profile; canthus rostralis rounded; loreal region oblique, slightly concave; nostril oval, protuberant, closer to tip of snout than eye; IND (2.9 mm) slightly narrower than IOD (3.0 mm) and wider than UEW (2.5 mm); eye large, horizontal diameter (ED 4.2 mm) equal to snout length (SL 4.2 mm); pupil oval, horizontal; pineal ocellus absent; tympanum distinct, diameter (TD 1.5 mm) smaller than half of ED; supratympanic fold distinct, extending from posterior corner of eye to above insertion of arm; vomerine teeth absent; tongue notched posteriorly; a pair of vocal sac slits on floor of mouth at both corners; an external subgular vocal sac.

Forelimb relatively robust; length of forearm and hand (FHL 14.1 mm) 47% of SVL; relative length of fingers I < II < IV < III; tips of all fingers expanded into discs with circummarginal grooves; disc of third finger large, slightly wider than tympanum; nuptial pads present on base of finger I; webbing between fingers rudimentary; subarticular tubercles prominent, rounded, single, formula 1, 1, 2, 2; supernumerary tubercles present; an inner metacarpal tubercle, oval; one outer metacarpal tubercle, rounded.

Heels overlapping when legs at right angle to body; tibiotarsal articulation reaching to middle of eye when hindlimb adpressed to body; relative length of toes I < II < III < V < IV; tips of toes expanded into discs with circummarginal grooves; discs of toes smaller than those of fingers; toes webbed, webbing formula I1.5–2II1.5–2.7III.5–3IV2.5–1.5V following Savage (1975); subarticular tubercles distinct and rounded, formula 1, 1, 2, 3, 2; supernumerary tubercles present; inner metatarsal tubercle oval; outer metatarsal tubercle absent.

Dorsal surface scattered with many small conical tubercles on head, upper eyelids, and dorsum; flanks of body and dorsal surface of limbs smooth, few small conical tubercles on hindlimbs and forearms; throat, chest, belly, and venter of thigh granulated; few small conical tubercles scattered on venter of thigh, tibia, and forearm.

#### Coloration of holotype.

**Figure 4.**
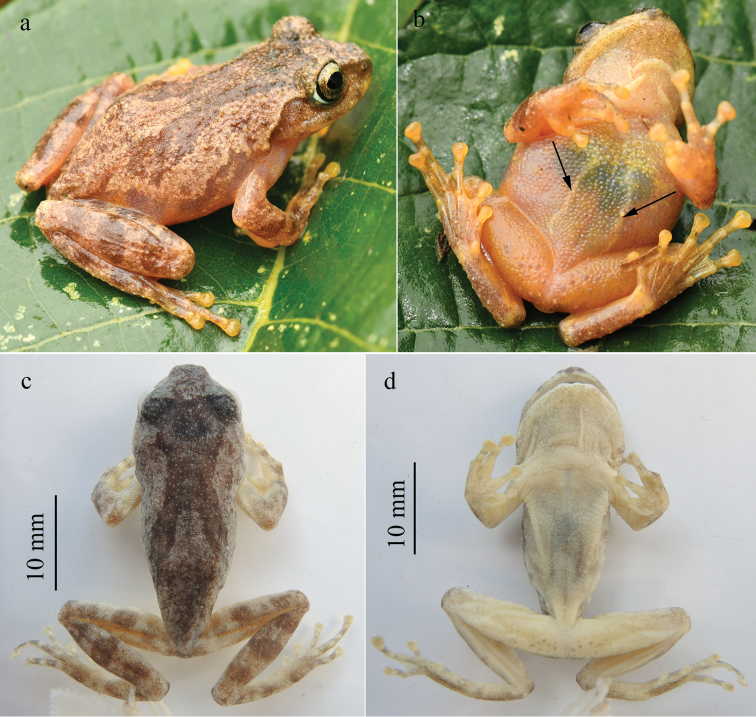
Dorsolateral (**a**) and ventral (**b**) views of the holotype of *Gracixalusyunnanensis* sp. n. in life and dorsal (**c**) and ventral (**d**) views of the holotype of *Gracixalusyunnanensis* sp. n. in preservative. Linea masculina is pointed by arrow.

In life, iris bronze; dorsal surface yellow brown with a dark brownish Y-shaped marking across back, covering interorbital region and posterior eyelids, bifurcating into two branches on the shoulder, and reaching the posterior of the back; limbs dorsally brown with dark brown bars; sides of head faint brown; flanks yellow brown, mottled with faint pink on lower part; minute dark spots densely scattered on lower part of flanks, temporal region, and upper jaw; skin of ventral surface semi-transparent, orangish with yellow spots; nuptial pads and discs faint yellow; linea masculina visible, white (Fig. 4b).

In preservative, color faded, pattern same as in life. Dorsal surface grayish brown, with a darker brown Y-shaped marking; dorsal side of limbs barred with dark brown; ventral surface of throat, chest, belly, forelimbs, and hindlimbs faded to whitish.

#### Morphological variation.

Measurements are shown in Table 3. Because the holotype and paratypes of the new species are all male, sexual dimorphism could not be determined. IOD is slightly wider than IND in holotype and most paratypes with the exception of KIZ 20160228, and TL is longer than FL in holotype and most paratypes with exceptions of KIZ 20160226 and KIZ 20160229.

Color of dorsal and ventral surfaces varied among individuals. Dorsal ground color of the holotype and four paratypes (KIZ 20160216, KIZ 20160223, KIZ 20160228, and KIZ 20160230) is yellow brown, and dorsal ground color of remaining paratypes (KIZ 20160226, KIZ 20160229, and GXNU YU000060) is red brown. Ventral surface of all specimens is nearly immaculate with the exception of paratype GXNU YU000060, which has dark marbling on throat, chest, and belly (Fig. 6). Conical tubercles on dorsum of specimens with red brown ground color are more distinct visually (Fig. 6).

**Figure 5.**
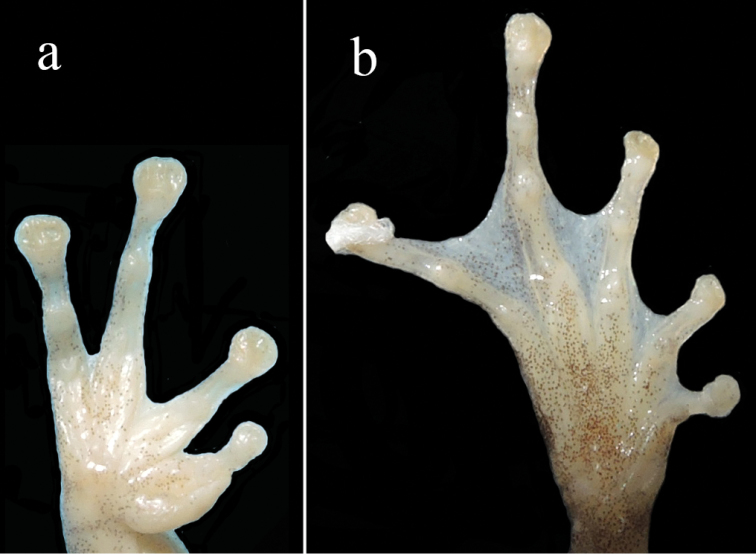
Ventral views of hand (**a**) and foot (**b**) of the holotype of *Gracixalusyunnanensis* sp. n. in preservative.

**Figure 6.**
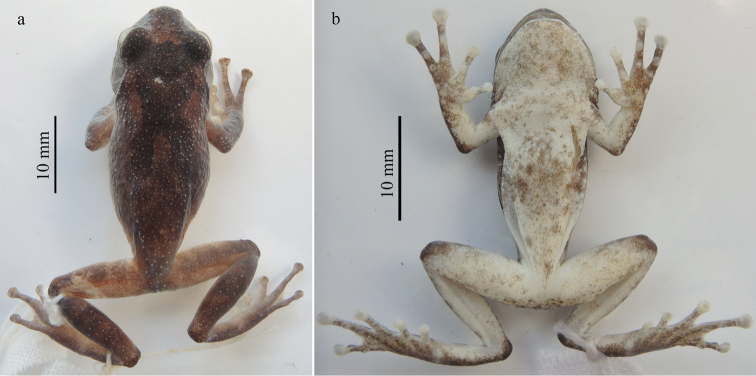
Dorsal view of paratype KIZ 20160226 (**a**) and ventral view of paratype GXNU YU000060 (**b**).

**Table 3.** Measurements (mm) of *Gracixalusyunnanensis* sp. n. Abbreviations defined in text.

Voucher no.	Sex	SVL	HL	HW	SL	IND	IOD	UEW	ED	TD	DNE	DNS	FHL	THL	TL	TFL	FL
KIZ 20160216	m	30.0	9.5	11.4	4.0	3.4	3.5	2.5	4.4	1.9	2.4	1.8	15.4	13.1	13.8	20.2	13.3
KIZ 20160222	m	29.7	9.5	10.9	4.2	2.9	3.0	2.5	4.2	1.5	2.4	1.7	14.1	12.3	13.2	18.8	12.9
KIZ 20160223	m	28.5	9.4	10.5	4.0	2.9	3.0	2.5	4.0	1.8	2.2	1.6	13.9	12.6	13.0	18.7	12.5
KIZ 20160226	m	34.2	10.1	12.1	4.7	3.3	3.8	2.8	4.4	1.9	2.5	2.1	15.6	13.3	14.1	21.1	14.2
KIZ 20160228	m	28.7	9.4	11.0	4.2	3.0	3.0	2.5	4.1	1.6	2.4	1.7	14.6	12.6	13.3	19.2	12.8
KIZ 20160229	m	26.0	9.0	9.4	3.5	2.6	2.8	2.3	3.5	1.3	2.0	1.3	12.8	11.0	11.6	17.0	11.6
KIZ 20160230	m	26.4	8.3	10.0	3.8	2.9	3.3	2.5	3.8	1.6	2.2	1.7	13.4	12.0	12.7	18.3	11.8
GXNU YU000060	m	27.3	8.7	9.5	4.1	2.7	2.8	2.5	3.7	1.7	2.3	1.7	13.4	11.9	12.7	18.8	12.3

#### Distribution.

In China, the new species is known from Yunnan (Lancang County, Menghai County, Lvchun County, and Jinping County). In addition, the new species also occurs in Laos (Houapan) and Vietnam (Lao Cai and Nghe An) because our molecular analyses revealed that samples from Houapan (KUHE 32453), Lao Cai (VNMN 4355, 4357, 4371), and Nghe An (AMS R173454) that were sequenced by previous studies also belong to the new species (Figs 2, 3). In Yunnan, specimens were found sitting on leaves of herbaceous plants (e.g., *Amomumtsaoko* and *Eupatoriumadenophorum*). No eggs and tadpoles were found.

#### Comparisons.

**Figure 7.**
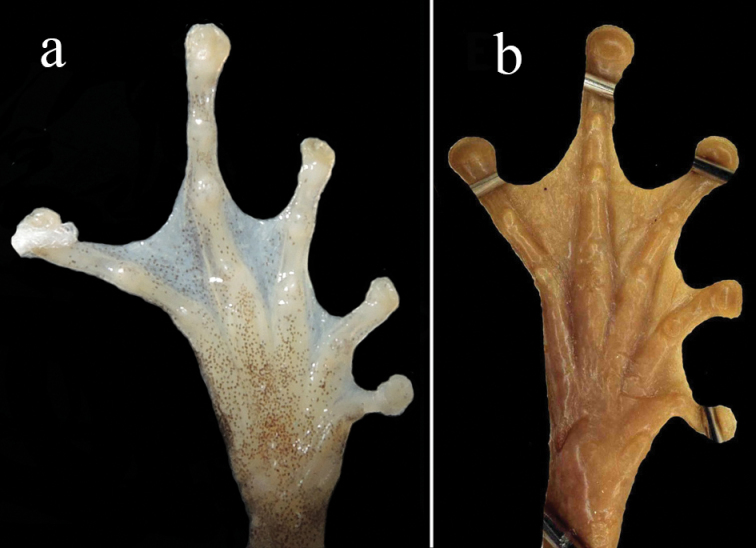
Ventral views of foot of the holotype of *Gracixalusyunnanensis* sp. n. (**a**) and lectotype of *Gracixaluscarinensis* (**b**; reproduced from Matsui et al. 2017).

A summary of morphological comparisons presents in Table 4. The new species can be distinguished from *G.ananjevae* by having distinctive conical tubercles on dorsum (versus absent), sides of body smooth (versus coarsely granular), skin of throat granular (versus plain), and snout rounded (versus slightly pointed); from *G.carinensis* by having smaller body size in males (SVL 26.0–34.2 mm versus 30.2–38.1 mm), having distinctive conical tubercles on dorsum (versus absent), having an external vocal sac in males (versus an internal vocal sac), ventral surface orangish (versus white), and less developed toe webbing (Fig. 7); from *G.gracilipes* by having bigger body size in males (SVL 26.0–34.2 mm versus 20–24 mm), distinctive conical tubercles present on dorsum (versus absent), dorsal surface yellow brown or red brown (versus greenish), males with an external vocal sac (versus internal), throat granular (versus smooth), finger webbing rudimentary (versus absent), tibiotarsal articulation reaching to eye (versus reaching to between eye and nostril), snout rounded (versus triangular pointed), white patch absent on temporal region (versus present), tibiotarsal projection absent (versus present), and iris bronze (versus brown); and from *G.guangdongensis* by having distinctive conical tubercles on dorsum (versus absent), dorsal surface yellow brown or red brown (versus brown), flanks nearly smooth with no black blotches on ventrolateral region (versus flanks rough, scattered with tubercles and black blotches on ventrolateral region), snout rounded (versus triangularly pointed), ventral surface orangish (versus throat and chest creamy white and belly light brown), and iris bronze (versus iris brown).

*Gracixalusyunnanensis* sp. n. differs from *G.jinggangensis* by having distinctive conical tubercles on dorsum (versus absent), flanks nearly smooth (versus rough with tubercles), snout rounded (versus triangularly pointed), ventral surface orangish and immaculate (versus throat and chest dirty white with dark specks, belly white anteriorly with dark marking and posteriorly yellowish), nuptial pads present only on finger I (versus nuptial pads present on fingers I and II), heels overlapping when hindlimbs held at right angles to the body (versus just meeting), and iris bronze (versus iris golden); from *G.jinxiuensis* by larger body size in males (SVL 26.0–34.2 mm versus 23.5–26.3 mm), males with an external vocal sac (versus vocal sac internal), flanks nearly smooth (versus rough with tubercles), linea masculina present (versus absent), ventral surface orangish and immaculate (versus ventral surface gray-brown with dark marbling), and sole of feet and palms smooth (versus rough with dense large tubercles); and from *G.lumarius* by smaller body size in males (SVL 26.0–34.2 mm versus 38.9–41.6 mm), dorsal surface yellow brown or red brown (versus yellow), and venter orangish and semi-transparent (versus venter opaque pink), supratympanic fold distinct (versus indistinct), and iris bronze (versus dark gold).

*Gracixalusyunnanensis* sp. n. can be distinguished from *G.medogensis* by having distinctive conical tubercles on dorsum (versus absent), dorsal surface yellow brown or red brown (versus grass green), males with an external vocal sac (versus an internal vocal sac), finger webbing rudimentary (versus absent), and venter orangish (versus pale green); from *G.nonggangensis* by having conical tubercles on dorsum (versus absent), dorsum yellow brown or red brown with a dark brown marking (versus yellowish-olive with a dark-green marking), males with an external vocal sac (versus internal), flanks smooth (versus rough with tubercles), finger webbing rudimentary (versus absent), linea masculina present in males (versus absent), tibiotarsal articulation reaching to eye (versus reaching to tip of snout), ventral surface immaculate (versus throat, chest, and belly white with dark marbling), nuptial pads present on finger I (versus absent), and iris bronze (versus olive); from *G.quangi* by having bigger body size in males (SVL 26.0–34.2 mm versus < 25 mm), dorsal surface yellow brown or red brown (versus olive-green), black spots absent on flanks and ventral surface of thighs (versus present), throat granular (versus smooth), finger webbing rudimentary (versus absent), snout rounded (versus triangular pointed), white patch absent on temporal region (versus present), tibiotarsal projection absent (versus present), and ventral surface orangish (versus opaque white with translucent pale green margins); and from *G.quyeti* by dorsal surface yellow brown or red brown (versus brownish to moss-green), flanks nearly smooth (versus rough with sharp tubercles), throat granular (versus smooth), tibiotarsal articulation reaching to eye (versus reaching to snout), supratympanic fold distinct (versus indistinct), and throat and chest immaculate (versus throat, margin of throat, and chest yellow-white with brown marbling).

*Gracixalusyunnanensis* sp. n. differs from *G.sapaensis* by having distinctive conical tubercles on dorsum (versus absent) and sides of body nearly smooth (versus coarsely scattered with large round tubercles); from *G.seesom* by bigger body size in males (SVL 26.0–34.2 mm versus 21.6–23.0 mm), conical tubercles present on dorsum (versus absent), dorsal surface yellow brown or red brown (versus tan), flanks nearly smooth with no white blotches (versus flanks with large tubercles and white blotches), throat granular (versus smooth), snout rounded (versus triangular pointed), nuptial pads present on finger I (versus absent), and iris bronze (versus golden); and from *G.supercornutus* by bigger body size in males (SVL 26.0–34.2 mm versus 22.0–24.1 mm), conical tubercles on dorsum small (versus considerable bigger horn-like projections in supraorbital area, around cloaca, and on dorsal surface, forelimbs and hindlimbs), dorsal surface yellow brown or red brown (versus greenish), snout rounded (versus triangular pointed), white patch absent on temporal region (versus present), and tibiotarsal projection absent (versus present).

The new species can be distinguished from *G.tianlinensis* by smaller body size in males (SVL 26.0–34.2 mm versus 30.3–35.9 mm), distinctive conical tubercles present on dorsum (versus absent), dorsal surface yellow brown or red brown (versus brown to beige), finger webbing rudimentary (versus absent), ventral surface orangish, immaculate, and semi-transparent (versus throat and chest gray with dark specks and belly creamy white, opaque), and nuptial pads present on finger I (versus on fingers I and II); and from *G.waza* by having distinctive conical tubercles on dorsum (versus absent), dorsal surface yellow brown or red brown (versus greyish-green to moss-green), throat granular (versus smooth), finger webbing rudimentary (versus absent), and ventral surface immaculate (versus throat and chest with dark marbling).

## Discussion

**Figure 8.**
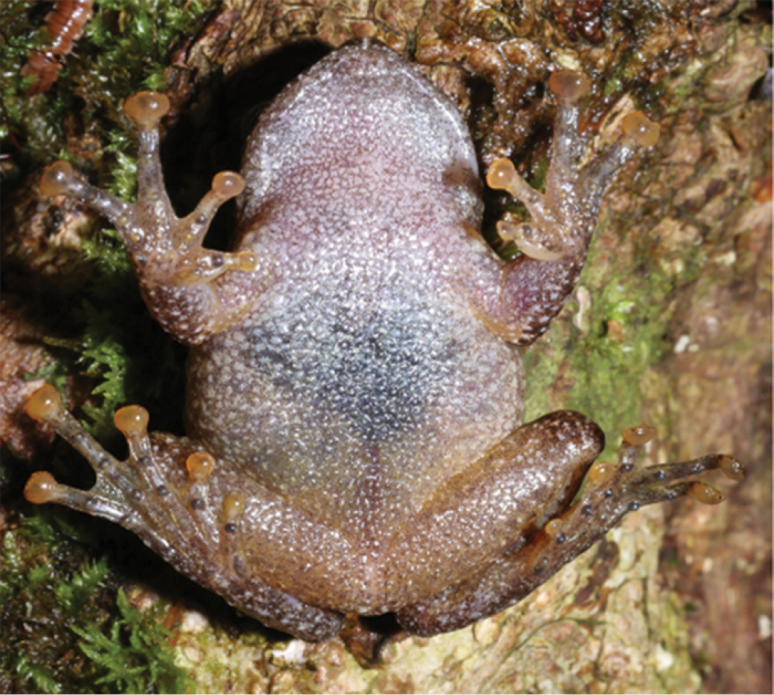
Ventral view of male holotype of *G.guangdongensis* (SYS a005724) in life (reproduced from Wang et al. 2018).

Although *G.yunnanensis* sp. n. only diverges from *G.guangdongensis* by a distance of 2.2%, it can be morphologically separated from *G.guangdongensis* by a series of characters including distinctive conical tubercles on dorsum (versus absent), dorsal surface yellow brown or red brown (versus brown), flanks nearly smooth with no black blotches on ventrolateral region (versus flanks rough, scattered with tubercles and black blotches on ventrolateral region), snout rounded (versus triangularly pointed), ventral surface orangish (versus throat and chest creamy white and belly light brown), and iris bronze (versus iris brown) (Table 4). In addition, the new species has linea masculina (Fig. 4b), whereas *G.guangdongensis* likely lacks linea masculina (Fig. 8), although it was not described in Wang et al. (2018). Moreover, the new species and *G.guangdongensis* were recovered as reciprocally monophyletic and the new species is not directly related to *G.guangdongensis* or other known congeners with strong support (Figs 2, 3). Therefore, we think that *G.yunnanensis* sp. n. should be diagnosed as an independent species. It has been revealed that low interspecific genetic distance seems to be very common in frogs from Southeast Asia (e.g., 2.2%−21.2% in Megophryidae, 1.8%−16.0% in Ranidae, and 1.5%−19.8% in Rhacophoridae; Grosjean et al. 2015).

Historically, *G.yunnanensis* sp. n. was once confused with *G.jinxiuensis* in that the Jinping specimen (KIZ 060821126), Houapan specimen (KUHE 32453), Nghe An specimen (AMS R173454), and Lao Cai specimens (VNMN 4355, 4357, 4371) were originally identified as *G.jinxiuensis* by Yu et al. (2008), Matsui et al. (2015), Rowley et al. (2011), and Matsui et al. (2017), respectively. However, the new species can easily be distinguished from *G.jinxiuensis* by having bigger body size, an external vocal sac, and linea masculina in males (Table 4).

*Gracixalus* now contains a total of 17 species and our phylogenetic analyses revealed that this genus consists of three major clades, one consisting of *G.lumarius* (Clade I), one consisting of *G.seesom*, *G.quyeti*, *G.quangi*, *G.supercornutus*, and *G.gracilipes* (Clade II), and one consisting of all other species (Clade III) (Figs 2, 3). This result is consistent with Zeng et al. (2017), Chen et al. (2018), and Wang et al. (2018). However, like these previous studies, the present study did not achieve a complete resolution of phylogenetic relationships among these three clades and phylogenetic relationships within clades II and III. Thus, more studies will be needed to resolve the phylogenetic relationships among this genus. Additionally, taxonomic confusions still exist in *Gracixalus*: Matsui et al. (2015) and Wang et al. (2018) considered that *G.nonggangensis* should be synonymized with *G.waza* because of low genetic distance between them. However, morphologically, males of *G.waza* have developed nuptial pads on finger I according to Nguyen et al. (2013), whereas males of *G.nonggongensis* lack nuptial pad according to Mo et al. (2013). If indeed this is the case, we would prefer that *G.nonggangensis* and *G.waza* represent two different species. Furthermore, cryptic species might exist in *G.nonggangensis* because its monophyly was not supported in Matsui et al. (2015) and Wang et al. (2018). In addition, studies will be necessary to confirm whether the specimen from Wenshan, Yunnan, China (voucher number: 03320Rao; GenBank accession no.: GQ285669) belongs to *G.ananjevae* or not. We found that they are sister to each other with strong support values (Figs 2, 3), which is consistent with Mo et al. (2013); the genetic distance between them is moderate (2.3%; Table 2).

**Table 4.** Morphological characters for comparisons among *Gracixalus* species. “?” = not known or not clearly defined in the literature.

Species	Adult male SVL (mm)	Conical tubercles on dorsum	Dorsal color in life	Vocal sac	Skin of body sides	Skin of throat	Finger webbing	Linea masculina	Tibiotarsal articulation	Snout	White patch on temporal region	Tibiotarsal projection	Supratympanic fold	venter	Nuptial pads	heels	iris
*G.yunnanensis* sp. n.	26.0–34.2	present, small	yellow brown or red brown	external	smooth, no black blotches	granular	rudimentary	present,	reaching eye	rounded	absent	absent	distinct	orangish with yellow spots, immaculate, semi-transparent	on finger I	overlapping	bronze
* G. ananjevae *	32	absent	?	?	coarsely granular	plain	rudimentary	?	reaching eye	slightly pointed	absent	absent	distinct	immaculate	on finger I	overlapping	?
* G. carinensis *	30.2–38.1	absent	purplish, reddish, or greyish brown	internal	?	granular	rudimentary	?	reaching eye	rounded	absent	absent	distinct	immaculate white	?	?	?
* G. gracilipes *	20–24	absent	greenish	internal	smooth with white stripe	smooth	rudimentary	?	reaching eye	triangularly pointed	absent	absent	distinct	yellowish white	on fingers I and II	overlapping	brown
* G. guangdongensis *	26.1–34.7	absent	brown	?	rough, black blotches	granular	absent	present	reaching between eye and nostril	triangularly pointed	present	present	distinct	throat and chest creamy white, belly light brown, semi-transparent	on finger I	overlapping	brown
* G. jinggangensis *	27.9–33.8	absent	brown to beige	?	rough with tubercles	granular	rudimentary	?	reaching eye	triangularly pointed	absent	absent	distinct	Throat and chest dirty white with dark specks, belly white anteriorly with dark marking and posteriorly yellowish, semi-transparent	on fingers I and II	just meeting	golden
* G. jinxiuensis *	23.5–26.3	?	brown	internal	rough with tubercles	granular	rudimentary	absent	reaching eye	rounded	absent	absent	distinct	gray-brown with dark marbling	on finger I	just meeting	?
* G. lumarius *	38.9–41.6	present	yellow	external	?	granular	rudimentary	?	?	rounded	absent	absent	indistinct	opaque pink	on finger I	?	dark gold
* G. medogensis *	26.5	absent	grass green	internal	?	granular	absent	present	reaching eye	rounded	absent	absent	distinct	pale green	on finger I	overlapping	?
* G. nonggangensis *	29.9–35.3	absent	yellowish-olive with dark-green mark	internal	rough with tubercles	granular	absent	absent	reaching tip of snout	rounded	absent	absent	distinct	white with dark marbling, semi-transparent	absent	overlapping	olive
* G. quangi *	< 25	present, small	olive-green	external	with black blotches	smooth	absent	?	?	triangularly pointed	present	present	distinct	opaque white with translucent pale green margins	on finger I	?	bronze
* G. quyeti *	?	present	brownish to moss-green	?	rough with sharp tubercles	smooth	rudimentary	?	reaching to snout	rounded	absent	absent	indistinct	belly immaculate white	?	overlapping	?
* G. sapaensis *	21–37	absent	Golden ochre	?	coarsely scattered with large tubercles	?	rudimentary	?	reaching eye	rounded	absent	absent	distinct	throat, chest, and belly light yellow, with dark marking	on finger I	overlapping	golden
* G. seesom *	21.6–23.0	absent	tan	?	with large tubercles and white blotches	smooth	rudimentary	?	reaching between eye and nostril	triangularly pointed	absent	absent	distinct	anterior belly opaque white and posterior belly translucent	absent	overlapping	golden
* G. supercornutus *	22.0–24.1	present, bigger horn-like	green with brown spots	?	?	granular	?	?	?	pointed	present	present	distinct	light with white spots	?	?	?
* G. tianlinensis *	30.3–35.9	absent	brown to beige	external	?	granular	absent	?	?	rounded	absent	absent	distinct	throat and chest gray with dark specks, belly creamy white, opaque	on fingers I and II	?	bronze
* G. waza *	27.1–32.9	absent	greyish-green to moss-green	?	with small granulars	smooth	absent	?	?	rounded	absent	absent	distinct	Throat and chest white with dark marbling, belly immaculate white, semi-transparent	on finger I	overlapping	?

## Supplementary Material

XML Treatment for Gracixalus yunnanensis
